# Associations and Association Meetings

**Published:** 1896-10

**Authors:** 


### ASSOCIATIONS AND ASSOCIATION MEETINGS.

Pennsylvania State V. M. A., F. S. Allen, Secretary, 800 N. 17th St., Philadelphia. Last meeting, Reading, Pa., October 6, 1896.

Schuylkill Valley V. M. A., U. G. Fredirici, Secretary, Tamaqua, Pa. Next meeting, Pottsville, Pa., December 16, 1896.

Lehigh Valley V. M. A.

Missouri Valley V. M. A., S. L. Hunter, Secretary, Ft. Leavenworth, Kan. Next meeting, Kansas City, Mo., October, 1896.

Missouri State V. M. A., T. A. Arnold, Secretary, Mexico, Mo. Last meeting, August 26, 1896.

New York State V. M. S., C. D. Morris, Secretary, Pawling, N. Y. Next meeting at Syracuse.

New York County V. M. A., R. W. Ellis, Secretary, 531 W. 152d St., New York City. Last meeting, Academy of Medicine, October 9, 1896.

New York German V. M. A., Edwin Aucker, Secretary, 167 W. 31st St., New York City.

Erie County V. M. S., C. J. Willganz, Secretary, 215 Carolina St., Buffalo, N. Y.

Oneida County V. M. S., J. M. Currie, Secretary, Oneida, N. Y. Next meeting, November 3, 1896.

Wyoming Valley V. M. A., H. R. Church, Secretary, Luzerne Boro, Pa, Last meeting, Montrose, July 31, 1896.

Wisconsin Society of V. G., W. G. Clark, Secretary, Beaver Dam, Wis. Semi-annual meeting, Milwaukee, subject to call of Secretary.

California State V. M. A., D. F. Fox, Secretary, Sacramento, Cal. Last meeting, September 9, 1896.

Niagara and Orleans Co. V. M. A., J. P. Thomson, Secretary, Niagara Falls, N. Y. Last meeting, Medina, N. Y., August 18, 1896.

New Hampshire V. M. A., L. Pope, Jr., Secretary, Portsmouth, N. H. Next meeting, December, 1896.

Maine V. M. A., W. L. West, Secretary, Ellsworth, Maine. Next meeting, Bangor, January, 1897.

Illinois State V. M. A., Albert Babb, Secretary, 724 E. Edwards St., Springfield, Ill. Next meeting, Chicago, November, 1896.

Ohio State V. M. A., W. H. Gribble, Secretary, Washington C. H., Ohio. Last meeting at Buffalo, N. Y., September, 1896.

Virginia State V. M. A., Thomas M. Sweeny, Secretary, Richmond, Va. Next meeting, Staunton, January 5, 1897.

Michigan State V. M. A., William Jopling, Secretary, 214 Main St., Owosso, Mich.

Iowa State V. M. A., J. E. Brown, Secretary, Oskaloosa, Iowa. Next meeting, Des Moines. Call of the President.

Western Iowa V. M. A., P. F. Gaunt, Secretary, Fort Dodge, Iowa.

V. M. A. of New Jersey, S. Lockwood, Secretary, Woodbridge, N. J. Last meeting, October 8, 1896.

New Jersey State V. M. A., A. T. Sellers, Secretary, 444 Benson St., Camden, N. J. Not in active existence.

Maryland State V. M. A., A. W. Clement, Secretary, 916 Cathedral St., Baltimore, Md.

Indiana Association of V. G., J. C. Rodger, Secretary, 30 W. 8th St. Ind. Next meeting , 1896.

Montreal V. M. A., Harri H. Dell, Secretary, Sullivan, Illinois. Last meeting, February 20, 1896.

Ontario V. M. Col. Soc., H. R. Ryder, Secretary, Delphi, N. Y.

Ontario V. M. A., C. H. Sweetapple, Secretary, Toronto, Ont.

North Dakota V. M. A., T. D. Hinebauch, Secretary, Fargo, N. Dakota.

U. S. V. M. A., S. Stewart, Secretary, 7½ S. James St., Kansas City, Mo. Next meeting, September, 1897.

Vet. Assoc. of Manitoba, W. J. Hinman, Secretary, Winnipeg, Manitoba.

Massachusetts V. M. A., H. P. Rogers, Secretary, 42 Gardner St., Boston, Mass. Meetings monthly.

Connecticut V. M. A., H. W. Eliot, Secretary, Ansonia, Conn.

Nebraska V. M. A., A. T. Everett, Secretary, S. Omaha, Neb. August, 1896, last meeting.

District of Columbia, H. W. Acheson, Secretary, foot of 26th and D St., N. W., Washington, D. C.

Philadelphia Soc. of V. Graduates, E. S. Muir, Secretary. Meets monthly.

Keystone V. M. A., W. L. Rhoads, Secretary, Lansdowne, Pa. Meets monthly.

